# Olfactory Performance as an Indicator for Protective Treatment Effects in an Animal Model of Neurodegeneration

**DOI:** 10.3389/fnint.2018.00035

**Published:** 2018-08-14

**Authors:** Anja Meyer, Anne Gläser, Anja U. Bräuer, Andreas Wree, Jörg Strotmann, Arndt Rolfs, Martin Witt

**Affiliations:** ^1^Institute of Anatomy, Rostock University Medical Center, Rostock, Germany; ^2^Research Group Anatomy, School of Medicine and Health Sciences, Carl von Ossietzky University Oldenburg, Oldenburg, Germany; ^3^Research Center for Neurosensory Science, Carl von Ossietzky University Oldenburg, Oldenburg, Germany; ^4^Institute of Physiology, University of Hohenheim, Stuttgart, Germany; ^5^Albrecht-Kossel-Institute for Neuroregeneration, Rostock University Medical Center, Rostock, Germany

**Keywords:** Niemann–Pick disease type C1, olfactory receptors, mouse model, astroglia, microglia, olfactory bulb, neurodegeneration, biomarker

## Abstract

**Background:** Neurodegenerative diseases are often accompanied by olfactory deficits. Here we use a rare neurovisceral lipid storage disorder, Niemann–Pick disease C1 (NPC1), to illustrate disease-specific dynamics of olfactory dysfunction and its reaction upon therapy. Previous findings in a transgenic mouse model (*NPC1^-/-^)* showed severe morphological and electrophysiological alterations of the olfactory epithelium (OE) and the olfactory bulb (OB) that ameliorated under therapy with combined 2-hydroxypropyl-ß-cyclodextrin (HPßCD)/allopregnanolone/miglustat or HPßCD alone.

**Methods:** A buried pellet test was conducted to assess olfactory performance. qPCR for olfactory key markers and several olfactory receptors was applied to determine if their expression was changed under treatment conditions. In order to investigate the cell dynamics of the OB, we determined proliferative and apoptotic activities using a bromodeoxyuridine (BrdU) protocol and caspase-3 (cas-3) activity. Further, we performed immunohistochemistry and western blotting for microglia (Iba1), astroglia (GFAP) and tyrosine hydroxylase (TH).

**Results:** The buried pellet test revealed a significant olfactory deterioration in *NPC1^-/-^* mice, which reverted to normal levels after treatment. At the OE level, mRNA for olfactory markers showed no changes; the mRNA level of classical olfactory receptor (ORs) was unaltered, that of unique ORs was reduced. In the OB of untreated *NPC1^-/-^* mice, BrdU and cas-3 data showed increased proliferation and apoptotic activity, respectively. At the protein level, Iba1 and GFAP in the OB indicated increased microgliosis and astrogliosis, which was prevented by treatment.

**Conclusion:** Due to the unique plasticity especially of peripheral olfactory components the results show a successful treatment in NPC1 condition with respect to normalization of olfaction. Unchanged mRNA levels for olfactory marker protein and distinct olfactory receptors indicate no effects in the OE in *NPC1^-/-^* mice. Olfactory deficits are thus likely due to central deficits at the level of the OB. Further studies are needed to examine if olfactory performance can also be changed at a later onset and interrupted treatment of the disease. Taken together, our results demonstrate that olfactory testing in patients with NPC1 may be successfully used as a biomarker during the monitoring of the treatment.

## Introduction

Apart from age, the most common physiological reason for neurodegeneration (Doty et al., 1984; Doty, 2018), accelerated neurodegeneration is often accompanied or preceded by olfactory deficits. For example, progressive olfactory deficits constitute early signs of idiopathic Parkinson’s disease (IPD), often years before first motor dysfunctions occur (Doty et al., 1988; Mesholam et al., 1998; Hawkes et al., 1999; Haehner et al., 2007; Djordjevic et al., 2008; Ross et al., 2008). After 4 years, 7% of individuals with olfactory loss have developed signs of IPD (Haehner et al., 2007). Patients with IPD, frontotemporal dementia and Alzheimer’s disease present elevated numbers of PG dopaminergic neurons (Huisman et al., 2004; Mundinano et al., 2011). Hyposmia has also been reported in several other conditions such as Huntington’s disease (Moberg et al., 1987; Barrios et al., 2007), amyotrophic lateral sclerosis (Günther et al., 2018), incidental Lewy body disease (Driver-Dunckley et al., 2014), the neurologic form of Gaucher’s disease (McNeill et al., 2012) and numerous more (for reviews see: Attems et al., 2015; Marin et al., 2018).

Olfactory impairment influences the patient’s quality of life in an increasingly aging society (Karpa et al., 2010; Croy et al., 2014), but investigations on the significance of olfactory dysfunction in neurodegenerative diseases, such as Niemann–Pick type C1 (NPC1), is usually not in the focus of research.

The exposed location of ORNs and the direct access to the CNS as well as the ability to lifelong adult neurogenesis make the olfactory system a starting point of neuropathologic events (Rey et al., 2018) and thereby an interesting research object. The olfactory system is unique in that it is by far the most proliferative CNS system harboring differentiating progenitor cells, which travel from the subventricular zone via the rostral migratory stream into the OB, where they differentiate into tyrosine hydroxylase (TH+) or GABA(+) interneurons (Alvarez-Buylla and Garcia-Verdugo, 2002; Doetsch, 2003; Lledo et al., 2006). The high central plasticity is accompanied by the high turnover of peripheral ORNs in the OE (Schwob, 2002; Mackay-Sim et al., 2015).

NPC1 is a rare autosomal-recessive lipid storage disease that is characterized by progressive neurodegeneration, inducing ataxia and impairment of intellectual function, as well as hepatosplenomegaly and dystonia (Vanier and Millat, 2003; Garver et al., 2007; Spiegel et al., 2009). The defect is caused by mutations in the *NPC1* gene which leads to disturbances in intracellular lipid trafficking and to accumulation of unesterified cholesterol, glycosphingolipids and other fatty acids in the endosomal/lysosomal system (Carstea et al., 1997). This impaired lipid transport leads particularly to an extensive loss of Purkinje cells in the cerebellum and degeneration of other central nervous compartments (Elleder et al., 1985; Tanaka et al., 1988; Sarna et al., 2003; Maass et al., 2015).

So far, there is no causal therapy of NPC1, though the iminosugar miglustat (Zavesca^®^) is the only approved drug in Europe used for supporting and symptomatic therapy in NPC1 (Patterson et al., 2007). Miglustat inhibits glucosylceramide synthase, a key enzyme of glycosphingolipid biosynthesis, reducing intracellular accumulation of metabolites, like sphingomyelin and sphingosine (Platt and Jeyakumar, 2008). The therapy results in delayed onset of neurological symptoms with increased lifespan (Patterson et al., 2007; Platt and Jeyakumar, 2008). Another promising therapy results further in prevention of cerebellar Purkinje cell loss, improved motor function and reduced intracellular lipid storage in *NPC1^-/-^* mice, caused by combination of miglustat, the neurosteroide allopregnanolone and HPßCD, a cyclic oligosaccharide (Davidson et al., 2009, 2016; Hovakimyan et al., 2013a; Maass et al., 2015). Interestingly, the exclusive administration of HPßCD results in reduced cholesterol storage in organs and causes later onset of neurological symptoms, furthermore confirmed by a clinical trial with *NPC1^-/-^* patients (Liu et al., 2010; Ramirez et al., 2010; Matsuo et al., 2013).

This study focuses on the investigation of the olfactory function in the NPC1 mouse model with different treatment strategies. Sensory malfunctions in NPC1 such as retina degeneration (Claudepierre et al., 2010; Yan et al., 2014) and hearing loss (King et al., 2014) have already been reported in an NPC1 mouse model. Using the same model, we previously reported a severe loss of ORNs in the OE as well as microgliosis and astrogliosis in the first central olfactory relay station, the OB (Hovakimyan et al., 2013b). These alterations can be largely prevented by combined treatment or monotherapy with HPßCD (Meyer et al., 2017).

In this report we show that the earlier observed neurodegeneration and cell proliferation at the peripheral level in NPC1 is also detectable in the OB, albeit at a somewhat lower degree. What is more, NPC1 condition in mice leads to olfactory impairment in a buried pellet test. Olfactory dysfunction is also accompanied by differential regulation in the expression of some olfactory receptors. Since structural and functional parameters tested can be almost completely reconstituted by both therapy arms applied in this study, we suggest that olfactory testing may be regarded as a biomarker during treatment monitoring.

## Materials and Methods

### Animals

Heterozygous breeding pairs of NPC1 mice (BALB/cNctr-Npc1m1N/-J) were obtained from Jackson Laboratories (Bar Harbor, ME, United States) for generating homozygous *NPC1*^-/-^ mutants and *NPC1^+/+^* control wild type mice. Mice were maintained under standard conditions with free access to food and water with a 12 h day/night cycle, a temperature of 22°C and a relative humidity of 60%. Genotypes were determined until postnatal day P7 by PCR analysis. This study was carried out in accordance with the recommendations of the German legislation on protection of animals and the Committee on the Ethics of Animal Experiments at the University of Rostock. The protocol was approved by the Landesamt für Landwirtschaft, Lebensmittelsicherheit und Fischerei Mecklenburg- Vorpommern (approval IDs: LALLF M-V/ TST/7221.3-1.1-011/16, and LALLF M-V/ TST/7221.3-1.1-030/12).

### Genotyping

For genotyping by PCR analysis, 1–2 mm of the tails were clipped at P6 and homogenized in DirectPCR-Tail and 1% proteinase K (Peqlab, Erlangen, Germany) at 55°C with 750 rpm for 16 h overnight on a Thermo Mixer (Eppendorf, Hamburg, Germany). Extracts were centrifuged for 30 s with 6000 rpm and PCR analysis was performed twice with 2 μl of the lysate and two different primer pairs under equal cycling conditions. For detecting the mutant allele (obtained fragment size 475 bp) primers 5′-ggtgctggacagccaagta-3′ and 5′-tgagcccaagcataactt-3′ and for the wild type allele (obtained fragment size 173 bp) 5′-tctcacagccacaagcttcc-3′ and 5′-ctgtagctcatctgccatcg-3′ were used.

### Pharmacologic Treatment

The following four groups were systematically evaluated: (1) Sham-treated *NPC1*^+/+^ (wild type) mice; (2) sham-treated *NPC1*^-/-^ mutant mice; (3) *NPC1*^-/-^ mutant mice, which received a combination therapy; (4) *NPC1*^-/-^ mutant mice, which received a HPßCD monotherapy.

We used two different therapeutic schedules for the *NPC1*^-/-^ mutants. The first one was a combination treatment of synergistically working drugs utilizing cyclodextrin, allopregnanolone and miglustat, starting at P7 with an injection of allopregnanolone (Pregnan-3alpha-ol-20-one; 25 mg/kg; Sigma Aldrich, St. Louis, MO, United States) dissolved in cyclodextrin (HPßCD; 2-hydroxypropyl-*β*-cyclodextrin; 4,000 mg/kg, i.p.; Sigma Aldrich, in Ringer’s solution) once a week, as described by Davidson et al. (2009). Additionally, 300 mg/kg miglustat (*N*-butyl-deoxynojirimycin; generous gift of Actelion Pharmaceuticals, Allschwil, Schwitzerland) dissolved in normal saline solution was injected i.p. daily from P10 to P22. Afterward, miglustat powder was administered mixed with food (therapeutic scheme in **Supplementary Figure S1**). For the second treatment schedule, allopregnanolone and miglustat were omitted and only HPßCD was injected weekly (monotherapy). Controls included *NPC1*^+/+^ animals as well as *NPC1*^-/-^ mutants, being untreated or received normal saline solution or Ringer’s solution without active substances (“sham-treated”). For a better understanding, sham-treated and untreated mice are designated as “sham-treated” in the following sections of this study, since previous studies did not demonstrate any differences between both groups (Schlegel et al., 2016).

### Buried Pellet Test

In order to verify whether the morphological alterations of the *NPC1*^-/-^ mice correlate with impaired olfactory ability we conducted an olfactory behavior test, the buried pellet test according to a protocol of Lehmkuhl et al. (2003). Forty-five *NPC1*^-/-^ and 19 *NPC1*^+/+^ control mice aged between P54-56 were tested. Briefly, mice got accustomed to a piece of sweetened cereal pellet (Honey Bsss Loops, Kellogg, Munich, Germany) 2 days prior to the test and during the fasting period 18–24 h before. On the day of the test mice were habituated to the testing environment for 1 h in a fresh cage with bedding. The testing cage was prepared with ∼ 3 cm bedding and one pellet was buried 0.5 cm below in one corner of the cage. The subject was placed in the test cage and the latency time was measured until the mouse uncovered the pellet. If a mouse did not find the pellet within the predetermined time of 300 s the experiment was terminated and a latency of 5 min was recorded.

Subsequently, the test was repeated using the same scheme but now the pellet was placed on the surface (surface pellet test) to exclude possible motor disorders or alterations in the food motivation (**Supplementary Figure S2**). Latencies are expressed as the mean ± SEM (**Supplementary Table S1**).

### 5-Bromo-2′-deoxyuridine (BrdU) Injections

BrdU is a thymidine analog, which is incorporated in DNA during the S-phase of DNA synthesis. Consequently, it is a reliable marker for the quantification of the proliferative potential of tissues (Dover and Patel, 1994; Onda et al., 1994; Winner et al., 2002). To label proliferating cells in the OB, 5–7 mice of either group were injected intraperitoneally with BrdU (solubilized in normal saline, 50 mg/kg, Sigma, St. Louis, MO, United States) twice a day from P40 to P46 as described earlier (Meyer et al., 2017). Additionally, a final single dose was given 1 h before perfusion at P55–56 for labeling the dividing cells of the OE (Meyer et al., 2017). Recent studies have noted that conventional dosage of BrdU may lead to considerable destruction of cells (Duque and Rakic, 2011). Thus, we cannot rule out a possible toxic effect upon application of BrdU, however, in our approach this potential error would be a systematic one that applies to all treated animal groups and is likely not to change the relative differences measured between groups.

### RNA Extraction and cDNA Synthesis

For qPCR analysis of olfactory receptor genes and olfactory markers, OE was dissected from 16 homozygous *NPC1^-/-^* mutants and 5 control wild type mice (*NPC1^+/+^*) of both sexes aged to 8 weeks and treated as described in “Pharmacologic Treatment.” Mice were deeply anesthetized with pentobarbital (90 mg/kg) and then decapitated. The dissected tissues were frozen in liquid nitrogen and stored at -80°C. TRIzol reagent (Thermo Fisher Scientific, Waltham, MA, United States) was used for homogenization of the tissue, followed by RNA extraction according to the manufacturer’s protocol. After precipitation and drying, RNA was resuspended in an aliquot of RNase and DNase-free water quantified by A_260nm_ spectrophotometry (BioSpectrometer^®^ basic, Eppendorf, Hamburg, Germany) and stored at -80°C. cDNA was synthesized with 5 μg of total RNA using the High-Capacity cDNA Reverse Transcription Kit (Thermo Fisher Scientific, Waltham, MA, United States) according to the manufacturer’s protocol. Control reactions were performed without MultiScribe Reverse Transcriptase. cDNA was stored at -20°C. The quality of amplified cDNA was controlled using *β-Actin* PCR.

### Quantitative Real-Time PCR (qRT-PCR)

Each PCR reaction contained 8 μl RNase and DNase-free water, 10 μl TaqMan^®^ Universal PCR Master Mix (Thermo Fisher Scientific, Waltham, MA, United States), 1 μl cDNA and 1 μl TaqMan Gene Expression Assays for transcripts (listed in **Supplementary Table S2**). mRNA of each sample was normalized relative to *Ppia* and *ß-Actin*, both of them have been proven as useful reference genes for quantitative RT-PCR (**Supplementary Figure S3**) (Kennedy et al., 2013). PCR thermocycling parameters were 95°C for 20 s and 45 cycles of 95°C for 1 s and 60°C for 20 s. For analysis of the relative change in gene expression we used the 2^-ΔCt^ method. The reactions were run on the CFX96 Touch^TM^ Real-Time PCR Detection System (Bio-Rad Laboratories, Hercules, CA, United States) using Bio-Rad CFX Manager 3.1 Software (Bio-Rad Laboratories, Hercules, CA, United States). Each value is the average of at least three separate experiments.

### Lysate Preparation and Western Blot

For the biochemical analysis *NPC1*^-/-^ mutants (*n* = 3) and *NPC1*^+/+^ wild type controls (*n* = 2) (of both sexes, aged to 8 weeks, were used for different therapeutic treatment schedules. Protein extracts were prepared from the OB of *NPC1^-/-^* mutants and *NPC1^+/+^* control mice (with different treatments as described in Section “Pharmacologic Treatment”). The tissues were frozen in liquid nitrogen and stored at -80°C. Lysate preparation and western blotting were performed according to Vierk et al. (2012) and Velmans et al. (2013) with slight modifications. Tissue was homogenized (POLYTRON^®^PT 3100 D, Kinematica, Luzern, Switzerland) in TRIzol reagent (Thermo Fisher Scientific, Waltham, MA, United States) followed by extraction of proteins according to the manufacturer’s instruction. Protein concentrations were determined with the Biospectrometer basic (Eppendorf, Hamburg, Germany). Homogenates were subjected to 10 or 12% SDS-PAGE under reduced conditions and subsequently transferred to a nitrocellulose membrane (Amersham Protran 0.45 NC, GE Healthcare, Boston, MA, United States). Blots were blocked for 1 h with 5 % BSA (TH, GFAP) or 5% non-fat dry milk (ß-Actin, Iba1) diluted in Tris-buffered saline (TBS) with 0.05% Tween^®^ 20 and incubated overnight at 4°C with the following antibodies: mouse anti-TH (1:1,000, MAB318, Merck, Darmstadt, Germany), mouse anti-GFAP (1:500, MAB360, Merck, Darmstadt, Germany), rabbit anti-Iba1 (1:200, 019-20001, Wako Pure Chemical Industries, Osaka, Japan) and mouse anti-ß-Actin (1:1,000, A5441, Sigma-Aldrich, St. Louis, MO, United States). Secondary antibodies were sheep anti-mouse IgG (1:5,000, GE Healthcare, Boston, MA, United States) and donkey anti-rabbit IgG (1:5,000, GE Healthcare, Boston, MA, United States) conjugated to horseradish peroxidase in 5% non-fat dry milk diluted in TBS with 0.05% Tween^®^ 20. After incubation for 1 h at room temperature proteins were detected using Pierce ECL Plus Western Blotting Substrate (Thermo Fisher Scientific, Waltham, MA, United States) and analyzed by using ImageLab 6.0 software (Bio-Rad Laboratories, Hercules, CA, United States).

### Immunohistochemistry

Nineteen *NPC1*^-/-^ mutants and 6 *NPC1*^+/+^ wild type controls of both sexes, aged to 8 weeks, were used for different therapeutic treatment schedules. Mice were deeply anesthetized with a mixture of 50 mg/kg ketamine hydrochloride (Bela-Pharm GmbH & Co KG, Vechta, Germany) and 2 mg/kg body weight of xylazine hydrochloride (Rompun; Bayer HealthCare, Leverkusen, Germany) and then intracardially perfused with normal saline solution, followed by 4% paraformaldehyde (PFA) in 0.1 M phosphate buffered saline (PBS). The animals were then decapitated, skinned, spare tissue was removed and the remaining skull including the nasal turbinates and the whole brain were post-fixed in 4% PFA for 24 h at 4°C. Subsequently, heads were decalcified in 10% EDTA for 5–6 days at 37°C, dehydrated and embedded in paraffin. The heads were serially cut in 10 μm in frontal direction from the tip of the nose to the caudal end of the OB and collected. For orientation, some sections were stained with routine hematoxylin & eosin (H&E).

For the quantification of proliferating cells every 10th section (spaced 100 μm apart) was subjected to anti-BrdU immunohistochemistry. Sections were deparaffinized, rehydrated and pretreated with microwaves in 0.1 M citrate buffer (5 min, 680 W) for antigen retrieval, followed by incubation with 3% hydrogen peroxide (H_2_O_2_) in PBS to block endogenous peroxidases for 30 min, and 5% normal goat serum (NGS) in PBS for 45 min to block non-specific binding sites. Subsequently, sections were incubated with primary antibody against rat BrdU (1:2,000, #OBT0030G, Abd Serotec, Puchheim, Germany) in 3% NGS/PBS overnight at 4°C. One section of each slide was used for negative control. After washing in PBS, the sections were sequentially incubated for 1 h with the secondary anti-rat IgG (1:200; Vector, Burlingame, CA, United States), the Avidin-biotin-complex (ABC) reagent for 1 h (Vectastain-Elite; Vector, Burlingame, CA, United States) and finally visualized with H_2_O_2_ – activated 3,-3′-diaminobenzidine (DAB, Sigma, Munich, Germany). Sections were dehydrated, mounted with DePeX and coverslipped.

In addition, immunohistochemical reactions against Iba1 (1:4,000, #019-19741, Wako, Osaka, Japan) and glial fibrillary acidic protein (GFAP, 1:2,000, #Z0334, Dako, Hamburg, Germany) were analyzed for the evaluation of microglial and astroglial reaction in the OB. For regeneration and plasticity activity of newborn neurons in the OE, we used Growth Associated Protein 43 (GAP43, 1:1,000, #EP890Y, Abcam, Cambridge, England). To observe differentiations in the number of PG interneurons we investigated the immunoreactivity of tyrosine hydroxylase (TH, 1:2,000, #AB1542, Millipore, Temecula, CA, United States). Apoptotic cells were labeled with anti-cleaved caspase-3 (cas-3, 1:500, clone Asp175, #9661, Cell Signaling Technology, Danvers, MA, United States). For controls, primary antisera were omitted. In control sections no reactivity was observed.

### Stereology and Quantification

Following Regensburger et al. (2009) and Sui et al. (2012) we quantified BrdU(+) and TH(+) cells of the unilateral OB in 2-7 sections per mouse with an interval of 200 and 100 μm, respectively, using an unbiased stereological method, the optical fractionator. For each group and each genotype 3–9 animals were counted using a computer-aided microscope (Olympus BX-51) and stereology software (Stereo Investigator v7.5, MBF Bioscience, Williston, ND, United States). The whole OB of one hemisphere was first outlined using a 2x or 4x objective lens. Counting was realized at 40x magnification. Cell densities of proliferating cells and TH(+) interneurons per mm^3^ of the OB were averaged and the four groups were compared. Therefore, the untreated mutants and untreated *NPC1*^+/+^ mice served as a reference for both, combination treated and HPßCD treated mice. Results are expressed as mean values ± SEM (**Supplementary Table S3**).

### Statistical Analysis

Statistical evaluation of the olfactory behavior test, the cell quantifications and the qRT-PCR was done with a non-parametric Mann-Whitney *U*-test by SPSS statistics 22/24 (IBM, Chicago, IL, United States) using genotype and treatment groups as independent variables. Graphs are created using GraphPad Prism 7. *p* ≤ 0.05 was considered significant.

## Results

### Treatment Prevents Smell Loss in *NPC1^-/-^* Mice

**FIGURE 1:**
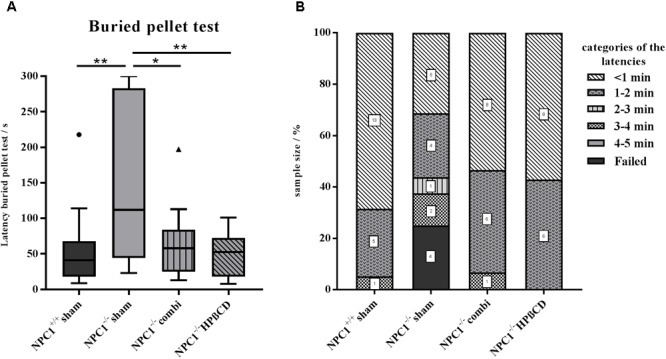
Results of the buried pellet test. Impaired sense of smell in *NPC1^-/-^* mice is restored after combination treatment and HPßCD monotherapy. **(A)** Performance of *NPC1*^+/+^ and differently treated *NPC1*^-/-^ mice: On average, sham-treated *NPC1*^-/-^ mice needed an almost 3-fold increased latency to find a buried piece of food, demonstrating a drastic loss of olfactory acuity. Both treatments, combination and HPßCD, normalized olfactory ability in *NPC1*^-/-^ mice to the level of *NPC1*^+/+^ controls. Box plot graphs represent the mean ± SEM and depict the median, the upper and lower quartiles, and outliers (circle and triangle). ^∗^*p* ≤ 0.05, ^∗∗^*p* ≤ 0.01 **(B)** Individual latencies of *NPC1*^+/+^ and differently treated *NPC1*^-/-^ mice: Results demonstrate a wide range of latencies. While 95% of the sham-treated *NPC1*^+/+^ mice uncovered the pellet within the first 2 min, only 56% of the sham-treated *NPC1*^-/-^ mice successfully finished within this time span and 25% even failed. After treatment, 93% of the combination and 100% of the HPßCD-treated *NPC1*^-/-^ mice finished within 120 s. Numbers in squares correspond to the number of mice that finished the test within this time span.

We used the buried pellet test for the evaluation of olfactory performance (**Figure 1** and **Supplementary Table S1**). Sham-treated *NPC1*^+/+^ mice needed on average 53 ± 12 s to uncover the pellet (**Figure 1A**). All sham-treated *NPC1*^+/+^ mice finished the test within the predetermined 5 min, whereby 95% of them finished within the first 60 s. In contrast, sham-treated *NPC1*^-/-^ mice needed significantly longer with an almost threefold latency of 145 ± 27 s (*p* = 0.003). Also, only 56% of the sham-treated *NPC1*^-/-^ mice finished within the first 60 s and 25% failed completely (**Figure 1B**). The remarkably increased latency of the sham *NPC1*^-/-^ animals was significantly reduced after a combination therapy with miglustat, allopregnanolone and HPßCD (*p* = 0.028). Combination-treated *NPC1*^-/-^ mice needed 64 ± 12 s to uncover the pellet, 93% finished within the first 120 s and none failed. The combination therapy significantly shortened the latency by 56% (81 s) compared with sham *NPC1*^-/-^ (*p* = 0.028). However, the latency is still 20% higher than the sham *NPC1*^+/+^ controls but without statistical proof (*p* = 0.331). HPßCD-treated *NPC1*^-/-^ mice behave similar to combination-treated *NPC1*^-/-^ mice. On average they needed 48 ± 8 s to find the buried pellet. All of them finished within the first 120 s. Consequently, the HPßCD treatment significantly reduced the latency by 68% (98 s) when compared with sham-treated *NPC1*^-/-^ mice (*p* = 0.007). With only 89% of the latency of the healthy controls, they are only slightly quicker than sham-treated *NPC1*^+/+^ mice (*p* = 0.898). To exclude motor disorders or alterations in the motivation for foraging of hungry mice, all subjects were tested a second time with the surface pellet test (**Supplementary Figure S2**). All mice of the 4 groups finished the surface pellet test within 60 s. The latency varied from minimum 1 s to a maximum of 47 s with mean values between 5.16 s (sham-treated *NPC1*^+/+^) and 11.07 s (HPßCD-treated *NPC1*^-/-^). Due to the very short latencies of the surface pellet test it can be assumed that the differences result from scattering of the measurements.

### Olfactory Receptors Are Differentially Regulated

**FIGURE 2:**
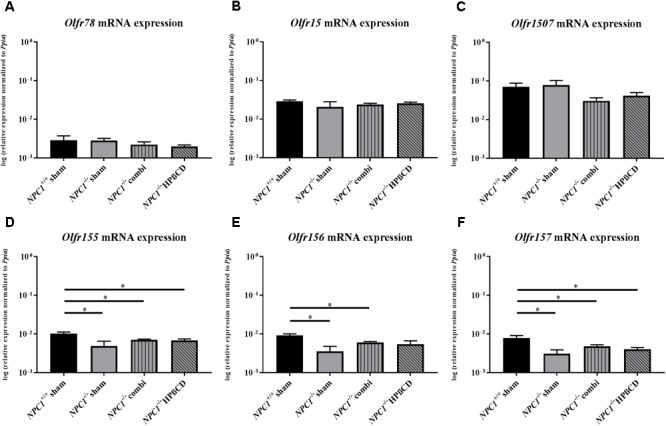
Olfactory receptor expression in the olfactory epithelium (OE) of *NPC1^-/-^* mutants. Quantitative RT-PCR exhibit no significant changes of the *Olfr78* **(A)**, *Olfr15* **(B)**, and O*lfr 1507* **(C)** expression level, in contrast to the *Olfr 155* **(D)**, *Olfr 156* **(E)**, and *Olfr 157* **(F)**, showed a significant reduction in sham-treated *NPC1^-/-^* mice compared to *NPC1^+/+^* mice. Combination treatment seemed to normalize this effect compared to *NPC1^-/-^* mice, nevertheless they demonstrated significantly lower expression levels compared to *NPC1^+/+^* controls. Additionally, *Olfr155* and *Olfr157* showed significantly lower expression levels after HPßCD treatment compared to *NPC1^+/+^* controls. Data are normalized to *Ppia* and represented as mean ± SEM, *n* = 3–4. ^∗^*p* ≤ 0.05.

In order to find possible connections between olfactory impairment in NPC1 disease and molecular events at the level of olfactory sensory neurons, we analyzed expression profiles of olfactory receptor genes (*Olfr)* located in 3 different zones of the mouse turbinate system (**Figure 2**; Nef et al., 1992; Strotmann et al., 1992, 1994; Ressler et al., 1993; Vassar et al., 1993; Sullivan et al., 1996).

*Olfr78*, located in ciliary membranes of ORNs in the dorsal zone of the OE (Conzelmann et al., 2000) exhibited no differences in sham-treated *NPC1*^-/-^ mice (0.0028 ± 0.0004) compared to *NPC1^+/+^* mice (0.0029 ± 0.0009) (*p* = 0.827), but combination (0.0022 ± 0.0004) (*p* = 0.127) and HPßCD (0.0020 ± 0.0002) (*p* = 0.275) treatment revealed a slight tendency of decreased expression compared to sham-treated *NPC1^-/-^* mice (0.0028 ± 0.0004; **Figure 2A**). The expression of *Olfr15*, located in the medial zone of the OE (Kaluza et al., 2004; Strotmann et al., 2004) showed no significant change, though a slightly decreased expression of sham-treated *NPC1*^-/-^ mice (0.0208 ± 0.0075) compared to sham-treated *NPC1^+/+^* mice (0.0291 ± 0.0022) (*p* = 0.386) was visible (**Figure 2B**).

*Olfr1507* is located in the lateral zone of the OE, showing no differences between *NPC1^+/+^* (0.0697 ± 0.0174) and sham-treated *NPC1^-/-^* mice (0.0777 ± 0.0247) (*p* = 0.773). However, both treatments lead to slightly decreased *Olfr1507* expression (combination treatment: 0.0305 ± 0.0061; HPßCD treatment: 0.0415 ± 0.0085) (**Figure 2C**).

*Olfr155, 156 and 157*, part of the same subfamily (OR37) are not broadly dispersed throughout the OE, but these receptors are concentrated in a small patch in the center of the OE (Strotmann et al., 1992, 1994, 1995, 2009; Kubick et al., 1997; Hoppe et al., 2006). Interestingly, the analysis shows significant changes of expression in the NPC1 mouse model (**Figures 2D–F**). *Olfr155* mRNA level in sham-treated *NPC1^-/-^* mice was significantly decreased (0.0049 ± 0.0016) compared to *NPC1^+/+^* mice (0.0103 ± 0.0010) (*p* = 0.043). Combination (0.0071 ± 0.0003) (*p* = 0.289) and HPßCD (0.0068 ± 0.0006) (*p* = 0.480) treatment normalized this decrease slightly, however, it was significantly decreased compared to *NPC1^+/+^* mice (combination: *p* = 0.034, HPßCD: *p* = 0.034). *Olfr156* exhibited similar changes with a significant decrease in sham-treated *NPC1^-/-^* mice (0.0035 ± 0.0012) compared to *NPC1^+/+^* mice (0.0092 ± 0.0009) (*p* = 0.020) that was slightly increased after combination (0.0060 ± 0.0004) (*p* = 0.289) and HPßCD (0.0054 ± 0.0012) (*p* = 0.289) treatments compared to *NPC1^-/-^* mice. Nevertheless, *Olfr156* expression of combination-treated *NPC1^-/-^* mice (*p* = 0.032) was significantly reduced compared to *NPC1^+/+^* mice. *Olfr157* showed similar changes as *Olfr155* and *156*. Sham-treated *NPC1^-/-^* mice (0.0031 ± 0.0008) exhibited equally decreased *Olfr157* expression compared to *NPC1^+/+^* mice (0.0078 ± 0.0013) (*p* = 0.043) that seems to be slightly increased after combination (0.0048 ± 0.0005) (*p* = 0.149) and HPßCD treatment (0.0040 ± 0.0004) (*p* = 0.248) compared to *NPC1^-/-^* mice, however, stayed significantly downregulated compared to *NPC1^+/+^* mice (combination: *p* = 0.043, HPßCD: *p* = 0.043).

### Initiation of Neurogenesis in the OE of *NPC1^-/-^* Mice

**FIGURE 3:**
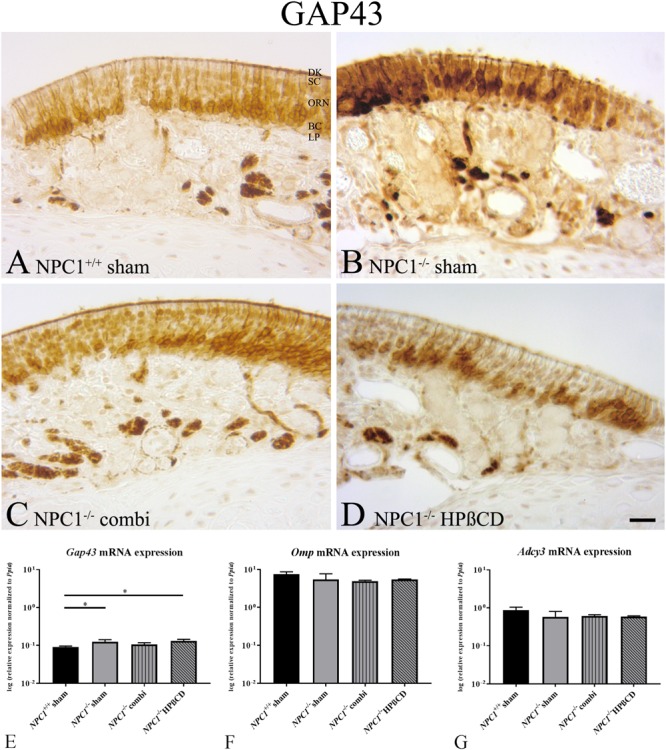
Increased neuronal regeneration in the olfactory epithelium (OE) of sham-treated *NPC1^-/-^* mice. **(A–D)** Immunohistochemical detection of GAP43 in the olfactory epithelium (OE). Newborn olfactory neurons were regularly distributed across the basal compartment of OE in *NPC1^+/+^* mice **(A)**, whereas they appeared more cluster-like dispersed in the OE’s middle layer of the other groups **(B–D)**. **(E)** Quantitative RT-PCR of the neuronal marker *Gap43* revealed increased expression in sham-treated *NPC1^-/-^* mice compared to *NPC1^+/+^* mice (*n* = 3). *Gap43* expression tended to be normalized in combination-treated *NPC1^-/-^* mice in contrast to HPßCD treatment that remained still significantly increased compared to sham-treated *NPC1^+/+^* mice. Expression analysis of *olfactory marker protein* (*Omp*, **F**) and *adenylate cyclase* (*Adcy3*, **G**) in the OE showed no significant changes (*n* = 3–4). Data are normalized to *Ppia* and represented as mean ± SEM. ^∗^*p* ≤ 0.05, scale bar in **(D)** = 20 μm.

In order to investigate the regenerative activity of ingrowing ORN, we performed immunohistochemistry and qPCR for Growth Associated Protein 43 (GAP43), a marker for newborn ORN in the OE (**Figure 3**).

Although not quantified, GAP43 immunohistochemistry did not seem to reveal differences in distribution and density of new ORN in any group investigated. ORN had a cluster-like distribution across the OE and showed a regular anatomy within the OE. However, it seems that GAP43 (+) ORN in *NPC1^-/-^* mice occupied more nuclei in the middle third of the OE than in each of the remaining groups (**Figure 3B**). Perikarya of GAP43(+) cells being ORN progenitors should be located closer to the basal membrane, as seen in controls and treated animals (**Figures 3A,C,D**).

*NPC1^-/-^* mice revealed a slight, but significant increase of *Gap43* mRNA (0.1233 ± 0.0179) compared to *NPC1^+/+^* mice (0.0911 ± 0.0047) (*p* = 0.05). This effect was not normalized after HPßCD treatment (0.1297 ± 0.0141) (*p* = 0.05) (**Figure 3E**). However, combination- treated *NPC1^-/-^* mice (0.1054 ± 0.0121) showed no significant regulation.

To evaluate the expression level of certain elements of the chemosensory signaling cascade during degeneration, expression of *Omp* was analyzed, albeit with no apparent change (**Figure 3F**). *Adcy3*, a cAMP-generating enzyme involved in the olfactory signal transduction cascade, along with *Omp* was not different between *NPC1^+/+^* and *NPC1^-/-^* mice (**Figure 3G**).

### Functional Histomorphology of the Olfactory Bulb

To identify possible reasons of impaired olfactory performance, we then studied the distribution of cellular markers at the immunohistochemical level and their expression at the molecular level in the OB.

**FIGURE 4:**
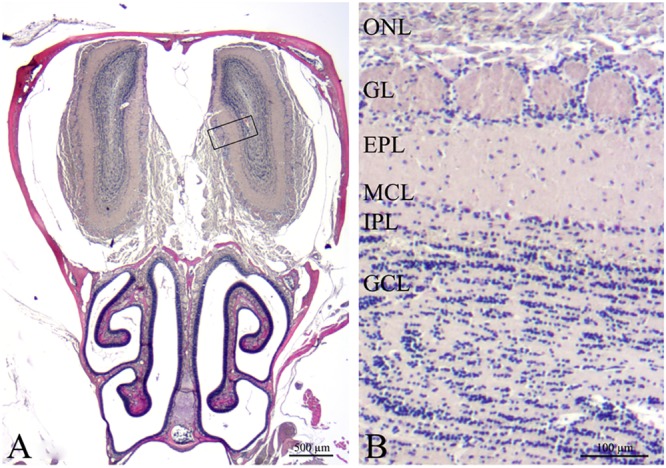
Histological overview of the peripheral and central olfactory structures (H&E). **(A)** Coronal section of the mouse anterior cerebral cavity that houses the olfactory bulbs and the nasal cavity including turbinates and septum with olfactory mucosa. The rectangle delimits the area magnified in **(B)**. Olfactory bulb with 6 layers: ONL, olfactory nerve layer; GL, glomerular layer; EPL, external plexiform layer; MCL, mitral cell layer; IPL, internal plexiform layer; GCL, granular cell layer. Scale bars = 500 μm **(A)**, 100 μm **(B)**.

Coronal sections of the OB were stained with H&E with an interval of 500 μm (**Figure 4**). Light microscopy did not show apparent morphological differences of the OB between any of the 4 groups.

#### Induction of Proliferation in the Olfactory System of *NPC1^-/-^* Mice

**FIGURE 5:**
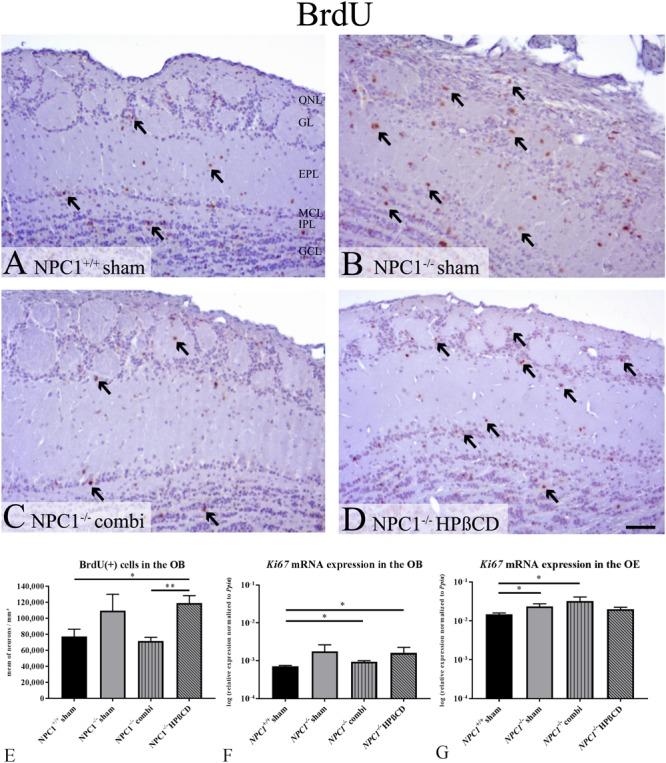
Increased proliferation in the olfactory system of *NPC1^-/-^* mice with different treatments. **(A–D)** Immunohistochemical reaction of BrdU(+) proliferating cells in the olfactory bulb (OB). **(A)** Sham-treated *NPC1*^+/+^ mice showed many newly formed cells in all layers of the OB. **(B)** The proliferation activity was visibly increased in sham-treated *NPC1*^-/-^ mice. This increase was reversible after combination treatment **(C)**, but not after HPßCD treatment **(D)**. **(E)** Quantification analysis of BrdU(+) cells in the unilateral OB demonstrated a strongly increased number of Brdu(+) cells in sham-treated *NPC1*^-/-^ mice, compared to sham-treated *NPC1*^+/+^ mice. Combination treatment reduced the proliferation activity in *NPC1*^-/-^ to the level of the controls. Surprisingly, HPßCD treatment did not notably decrease the proliferation density in *NPC1*^-/-^. **(F)** Quantitative RT-PCR of differently treated *NPC1^-/-^* mice in comparison with sham-treated *NPC1^+/+^* mice revealed an increased *Ki67* mRNA expression in the OB (*n* = 4) and **(G)** in the OE (*n* = 3–4). Data are normalized to *Ppia* and represented as mean ± SEM. ^∗^*p* ≤ 0.05, ^∗∗^*p* ≤ 0.01, scale bars **(A–D)** = 50 μm.

Earlier investigations of the proliferation activity in the OE of *NPC1*^-/-^ mice proved a notable increase of newly formed cells particularly after therapy (Meyer et al., 2017). Based on these findings, we further evaluated the proliferation activity of the OB. In sham-treated *NPC1*^+/+^ mice, most BrdU(+), proliferating cells were observed in the granular cell layer (GCL), fewer were detectable in the GL, the EPL, the MCL and the IPL (**Figure 5A**). In sham-treated *NPC1*^-/-^ mice an increase in the number of BrdU(+) cells in all layers became evident (**Figure 5B**). This increase seemed reversible after combination treatment (**Figure 5C**) and is contrasted by the outcome in HPßCD-treated *NPC1*^-/-^ mice, where this reconstitution of increased BrdU(+) immunoreactivity could not be observed; the number of BrdU(+) cells in all layers of the OB remained at an increased level (**Figure 5D**).

For a quantitative assessment of the proliferation activity, we counted the BrdU(+) cells in one bulb of each individual (**Figure 5E** and **Supplementary Table S3**). In the OB of sham-treated *NPC1*^+/+^ mice an average density of 77,327 ± 9,109 cells per mm^3^ was determined. In sham-treated *NPC1*^-/-^ mice this number increased to 109,557 ± 20,446 BrdU(+) cells per mm^3^ (41.7% higher compared to the healthy controls; *p* = 0.253). Interestingly, in combination-treated *NPC1*^-/-^ mice, a density of 71,779 ± 4,405 cells/ mm^3^ was found, a reduction by 34.5% (*p* = 0.291). With a small deviation of only 7.2% the proliferation went down almost to the normal level of the *NPC1*^+/+^ controls (*p* = 0.584). Surprisingly, in contrast to the combination therapy, the monotherapy with HPßCD did not lead to a notable decrease in the density of the proliferating cells (118,954 ± 9,298 cells/ mm^3^) in *NPC1*^-/-^ mice. In fact, this represents a significant enhancement of 54% when compared to sham-treated *NPC1*^+/+^ mice (*p* = 0.032) and even a slight, although not significant increase (*p* = 0.565) compared to combination-treated *NPC1*^-/-^ mice. The difference in the percentage of proliferating cells between combination- and HPßCD-treated *NPC1*^-/-^ mice is highly significant (*p* = 0.004), with 65.7% less BrdU(+) cells in combination-treated *NPC1*^-/-^, indicating a noticeable difference between both therapies.

Based on the above-mentioned results at the cellular level we performed qRT-PCR of the proliferation marker Ki67 (**Figure 5F**). qRT-PCR displayed the total *Ki67* mRNA in the OB normalized to the housekeeping gene *Ppia*. Generally, proliferating cells showed an increase of *Ki67*. Our analysis (*n* = 4) suggests the tendency of increased *Ki67* expression in sham-treated *NPC1^-/-^* mice (0.0018 ± 0.0009) compared to *NPC1^+/+^* mice (0.0007 ± 0.00004) (*p* = 0.149). Combination treatment of *NPC1^-/-^* mice showed a slightly reduced *Ki67* expression (0.009 ± 0.00007) (*p* = 0.564), but still increased compared to *NPC1^+/+^* mice (*p* = 0.021). HPßCD treatment (0.0016 ± 0.0007) revealed similar expression as sham-treated *NPC1^-/-^* mice (*p* = 1.0) and significantly increased expression compared to *NPC1^+/+^* mice (*p* = 0.021). There was no significant difference between HPßCD and combination treatment (*p* = 1.0), however, the latter seems to be more efficient to decrease mRNA expression. In summary, *Ki67* expression analysis of the OB supports the results of the BrdU quantification. In order to achieve complimentary data for earlier BrdU analysis of olfactory epithelial cells (Meyer et al., 2017) we also performed a *Ki67* expression analysis of the OE and observed an about 20-fold increase of *Ki67* expression compared to OB (**Figure 5G**). *Ki67* expression in the OE was significantly increased in sham- treated *NPC1^-/-^* mice (0.0236 ± 0.0039) compared to *NPC1^+/+^* mice (0.0148 ± 0.0012) (*p* = 0.021). This regulation was enhanced with combination treatment of *NPC1^-/-^* mice (0.0322 ± 0.0090) (*p* = 0.034). HPßCD treatment slightly increased expression (0.0199 ± 0.0023) compared to *NPC1^+/+^* mice (*p* = 0.157). Both, HPßCD (*p* = 0.048) and combination (*p* = 0.480) treatments showed no significant change in comparison with sham-treated *NCP1^-/-^* mice.

#### Increased Apoptotic Activity in the OB of *NPC1^-/-^* Mice

**FIGURE 6:**
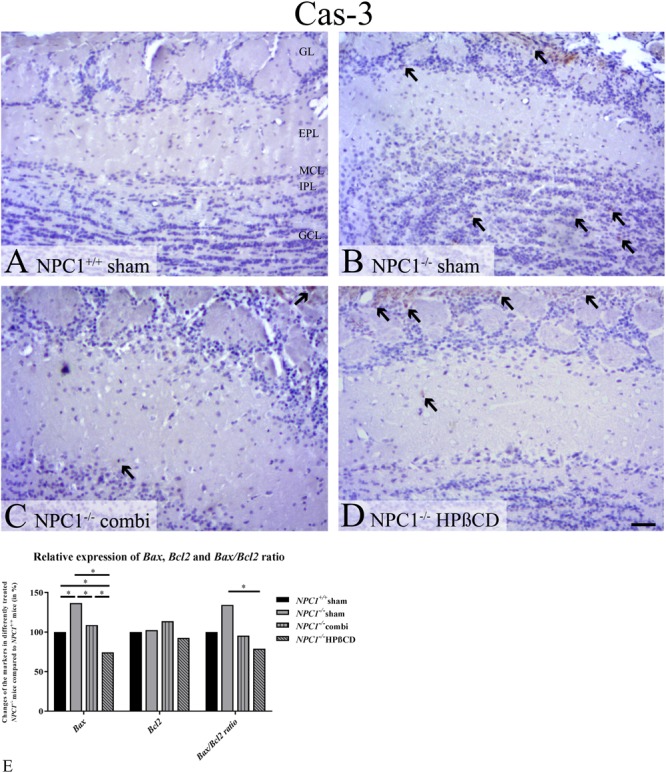
Increased number of apoptotic cells in the olfactory bulb (OB) of *NPC1^-/-^* mice confirmed by quantitative RT-PCR. **(A–D)** Immunohistochemical reaction of cas-3(+) apoptotic cells in the olfactory bulb (OB). **(A)** Sham-treated *NPC1*^+/+^ mice showed only very few apoptotic cells. **(B)** Sham-treated *NPC1*^-/-^ demonstrated a clear increase of apoptotic cells, mainly in the granular cell layer (GCL). Both, combination **(C)** and HPßCD **(D)** treatments reduced apoptotic activity in *NPC1^-/-^* mice. **(E)** Changes of *Bax* and *Bcl2* mRNA expression including *Bax/Bcl2* ratio, in the OB (*n* = 3) of differently treated *NPC1^-/-^* mice compared to *NPC1^+/+^* mice. The apoptotic marker *Bax* exhibited a significant upregulation of sham-treated *NPC1^-/-^* mice compared to *NPC1^+/+^* mice, that was significantly reduced after combination and HPßCD treatments. Even HPßCD treatment reduced the expression significantly in comparison with *NPC1^+/+^* mice. Here, expression of *Bcl2*, an anti-apoptotic protein, remained unchanged. The *Bax/Bcl2* ratio showed a significant upregulation in sham-treated *NPC1^-/-^* mice compared to HPßCD-treated *NPC1^-/-^* mice. Data (*n* = 3) are represented as mean (%) compared to sham-treated *NPC1^+/+^* (set at 100%), ^∗^*p* ≤ 0.05, scale bars **(A–D)** = 50 μm.

Caspase-3- positive cells [Cas-3(+)] occurred only rarely in the OB of sham-treated *NPC1*^+/+^ mice. In contrast, the OB of sham-treated *NPC1*^-/-^ contained numerous apoptotic cells, mainly in the GCL. Both, combination as well as HPßCD treatments led to a reduction of apoptotic cells in *NPC1*^-/-^ mice, Cas-3(+) cells were found mainly in the MCL and GL (**Figures 6A–D**).

In order to confirm the results of Cas-3 immunoreactivity we performed expression analysis via qRT-PCR using the apoptotic markers *Bax* and *Bcl2* (**Figure 6E** and **Supplementary Table S4**). *Bax*, an apoptotic activator, increased significantly in *NPC1^-/-^* mice (0.0470 ± 0.0030) by 36.7% compared to *NPC1^+/+^* mice (0.0344 ± 0.0015) (*p* = 0.05). Combination treatment (0.0374 ± 0.0023) decreased this apoptotic effect by 27.7% (*p* = 0.05), whereas HPßCD treatment (0.0256 ± 0.0043) reduced the expression by 62.3% (*p* = 0.05) compared to sham-treated *NPC1^-/-^* mice. The HPßCD treatment reduced the *Bax* expression by 34.5% more than combination treatment that exhibited even a 25.6% less expression than *NPC1^+/+^* (*p* = 0.05).

Expression of *Bcl2*, an apoptotic suppressor, was not significantly changed in any of the groups. Only combination-treated *NPC1^-/-^* mice tended to show increased *Bcl2* expression by around 13% compared to *NPC^+/+^* and *NPC1^-/-^* mice. HPßCD treatment exhibited slightly reduced mRNA expression (7.4%).

The ratio of *Bax* and *Bcl2* serves as an indicator of cell susceptibility to apoptosis and is correlated with the progression of several diseases (Oltvai et al., 1993; Korsmeyer, 1999; Scopa et al., 2001; Salakou et al., 2007). The *Bax/Bcl2* ratio in NPC1 was increased by 34.5% compared to controls. The combination treatment reduced the expression by 38.9%, but only HPßCD treatment decreased the expression in *NCP1^-/-^* mice significantly by around 55.4%, which was even 21% less than controls reveal. Summarizing, the *Bax/Bcl2* ratio suggests increased cell susceptibility to apoptosis that may be reduced via HPßCD treatment.

#### Enhanced Glia Cell Activation in *NPC1^-/-^* OB

**FIGURE 7:**
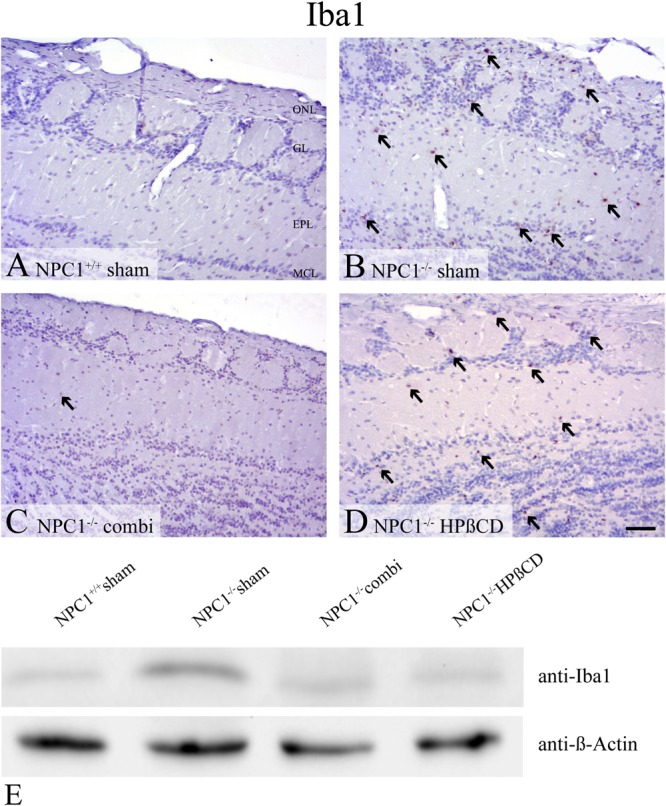
Pronounced microgliosis in the olfactory bulb (OB) of *NPC1^-/-^* mice, reduced by combination treatment. **(A–D).** Immunohistochemical reaction of Iba1(+) microglial cells in the OB. **(A)** Sham-treated *NPC1*^+/+^ mice revealed no reactivity for Iba1. **(B)** Sham-treated *NPC1*^-/-^ demonstrated a noticeable increase of Iba1(+) cells in the granular cell layer (GCL), external plexiform layer (EPL) and glomerular layer (GL). The microgliosis was reduced after combination treatment in *NPC1*^-/-^ mice **(C)**. However, HPßCD treatment retained an elevated microglial activity in *NPC1*^-/-^ mice **(D)**. Western blot analysis of the OB of differently treated *NPC1^-/-^* mice compared to sham-treated *NPC1^+/+^* mice **(E)**. Membrane was probed with an anti-Iba1 antibody and showed a clearly increased Iba1 signal (∼17 kDa) in sham-treated *NPC1^-/-^* mice compared to *NPC1*^+/+^ mice. The combination and HPßCD treatments revealed reduced microgliosis compared to sham-treated *NPC1^-/-^* mice. ß-Actin (∼42 kDa) was used as loading control.

Iba1, a marker of microglia that is associated with inflammatory processes in neurodegenerative diseases, revealed no reactivity in OB of sham-treated *NPC1*^+/+^ mice (**Figures 7A–D**). However, sham-treated *NPC1*^-/-^ mice demonstrated a noticeable increase of Iba1(+) cells in the GCL, EPL and GL of the OB. Western blot analysis confirmed the increase of Iba1 in sham-treated *NPC1^-/-^* OB (**Figure 7E**). The microgliosis was strongly reduced after combination therapy as revealed by immunohistochemistry and western blot. A monotherapy with HPßCD alone had no effect on the microglia immunoreactivity compared to *NPC1^-/-^* mice (**Figure 7D**).

**FIGURE 8:**
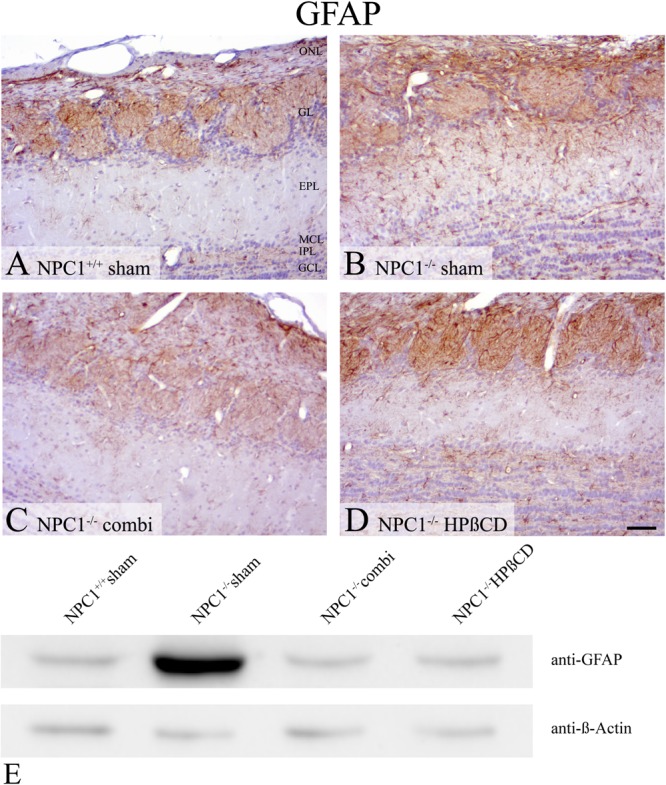
Astrogliosis of sham-treated *NPC1^-/-^* mice is increased in *NPC1^-/-^* mice and reduced after combination and HPßCD treatment. **(A–D)** Immunohistochemical reaction of GFAP in the OB. **(A)** Sham-treated *NPC1*^+/+^ mice revealed a balanced distribution pattern of astrocytes in all layers of the OB, mainly in the olfactory nerve layer (ONL), glomerular layer (GL) and granular cell layer (GCL). **(B)** In contrast, sham-treated *NPC1*^-/-^ demonstrated a pronounced astrogliosis that was reduced after combination (combi) **(C)** and HPßCD **(D)** treatments. **(E)** Western blot from total protein lysates of the OB from differently treated *NPC1^-/-^* mice compared to sham-treated *NPC1^+/+^* mice. To detect GFAP protein (∼59 kDa) membrane was probed with anti-GFAP antibody that confirms the distinct increase of GFAP(+) cells from sham-treated *NPC1^-/-^* mice. Combination and HPßCD treatments showed reduced astrogliosis confirming immunohistochemical results. ß-Actin (∼ 42 kDa) was used as loading control.

Preceding studies also revealed an intense immunoreactivity for the astroglial marker GFAP in *NPC1*^-/-^ mice that is, similar to Iba1, involved in neuropathological changes (Hovakimyan et al., 2013b; Meyer et al., 2017). In sham-treated *NPC1*^-/-^ mice, GFAP immunohistochemistry (**Figures 8A–D**) demonstrated a balanced distribution pattern of astrocytes in all layers of the OB, whereby the ONL, GL and the GCL stand out clearly against the EPL. In contrast, sham-treated *NPC1*^-/-^ mice showed a pronounced astrogliosis resulting in hardly definable layers of the OB. The distinct increase is confirmed via western blot analysis of sham-treated *NPC1^-/-^* OB compared to *NPC1^+/+^* OB (**Figure 8E**). This finding was remarkably reduced after both, combination and HPßCD treatments shown by immunohistochemistry and western blotting.

#### Tyrosine Hydroxylase Protein Level Is Reduced in *NPC1^-/-^* OB

**FIGURE 9:**
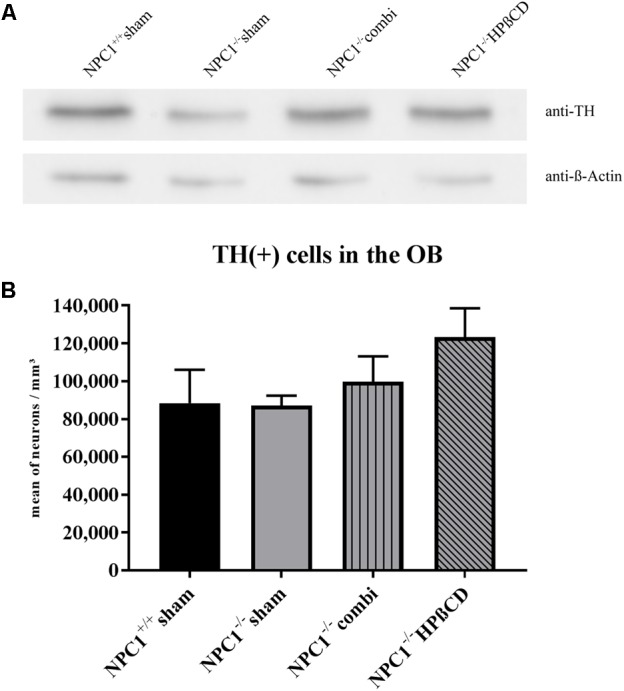
Reduced TH protein in *NPC1^-/-^* OB is not induced by loss of TH(+) periglomerular neurons (PG). **(A)** Western blot from total protein lysates of the OB from differently treated *NPC1^-/-^* mice compared to sham-treated *NPC1^+/+^* mice. To detect the TH protein (∼59 kDa) membrane fraction was probed with anti-TH antibody that showed reduced TH in sham-treated *NPC1^-/-^* mice compared to *NPC1^+/+^* mice. Both, combination and HPßCD treatments normalized this reduction. ß-Actin (∼42 kDa) was used as loading control. **(B)** Analysis of TH(+) dopaminergic neurons in the OB. Sham-treated *NPC1*^-/-^ mice showed no alterations in the number of TH(+) periglomerular neurons (PG) when compared to sham-treated *NPC1*^+/+^ control mice. Considering the results of the western blot analysis it suggests a reduction of TH/PG cell. Both treatments, combination and HPßCD, slightly increased the number of TH(+) PG in *NPC1*^-/-^ mice. However, combination therapy had a smaller therapy-induced influence on the TH(+) immunoreactivity in *NPC1*^-/-^ mice. All data represent the mean ± SEM.

Based on the massive loss of ORN in the OE of *NPC1*^-/-^ mice (Meyer et al., 2017), we investigated TH immunoreactivity in dopaminergic PG neurons of the OB (**Figure 9**). Western blot analysis showed a slight decrease of TH in *NPC1^-/-^* OB compared to the *NPC1^+/+^* control that seems to be normalized after combination and HPßCD treatment (**Figure 9A**). Determining whether this regulation was induced by an altered number of TH(+) cells we performed a quantitative analysis, counting TH(+) PG cells (**Figure 9B** and **Supplementary Table S3**). In sham-treated *NPC1*^+/+^ mice an average of 88,419 ± 17,605 TH(+) cells/mm^3^ was determined. With 87,093 ± 5,202 cells/mm^3^ (98.5 %) the number of TH(+) PG cells remained unchanged in sham-treated *NPC1*^-/-^ mice. Compared to the density of sham-treated *NPC1*^+/+^ mice, an increase was found in combination-treated *NPC1*^-/-^ mice (99,714 ± 13,380 TH(+) cells/mm^3^; 12.8 %) as well as in HPßCD-treated *NPC1*^-/-^ mice, with 123,253 ± 15,234 cells/mm^3^ (39.4%). However, the increased density of TH(+) PG cells after HPßCD treatment of *NPC1*^-/-^ mice was not statistically significant (*p* = 0.127). Although both therapies contained HPßCD, the combination therapy had a smaller influence on the density of TH(+) interneurons. Thus, these results suggest that the regulation of TH protein in *NPC1^-/-^* mice (**Figure 9A**) was not induced by alterations in the number of TH(+) cells, but rather by a reduction of TH protein/PG cell.

## Discussion

This paper addressed the issue of olfactory performance in a rare neurodegenerative disease, Niemann–Pick Type C1 (NPC1). Earlier investigation of our group has shown that significant olfactory degeneration occurs at the structural level (Hovakimyan et al., 2013b). This is accompanied by a remarkable increase of proliferation in the OE that can both be halted by pharmacologic treatment (Meyer et al., 2017). Therefore, the main motivation for this paper was to demonstrate that olfactory testing may be used as a suitable biomarker to evaluate the course of the neurodegenerative signs and symptoms of NPC1. Furthermore, we investigated the impact of the different treatment strategies on structural and physiological functions. Improved olfactory performance during the course of the therapy suggests that early treatment of NPC1 disease rescues olfactory function.

### Decreased Olfactory Function in *NPC1*^-/-^ Mice

Using a simple olfactory screening test we showed that *NPC1*^-/-^ mice actually needed significantly more time to find buried pellets in their bedding and that this impairment could be prevented by pharmacologic treatment.

As expected, *NPC1*^-/-^ mice had a severe smell deficit compared to healthy control mice, particularly an almost 3-fold increased latency to find a buried piece of food. The results of the buried pellet test, however, showed a wide range of latencies. While 25% of the sham-treated *NPC1*^-/-^ did not succeed to find the food pellet within a predetermined time of 5 min, 31% were as fast as the mean of sham-treated *NPC1*^+/+^ mice; the results indicate that the severe morphological damages can in some individuals be compensated during the complex process of olfaction. In a control surface pellet test *NPC1*^-/-^ mice had no difficulties finding an exposed piece of food indicating that they most likely have no impairments of motor skills or an altered motivation for foraging. By this, we confirmed and extended the results of Seo et al. (2014) who also demonstrated a poor olfactory performance in *NPC1*^-/-^ mice. Also, our own recent studies using electro-olfactogram recordings from the olfactory mucosa revealed a tendency of decreased odor induced response amplitudes in *NPC1*^-/-^ mice (Hovakimyan et al., 2013b).

### Differential Regulation of Olfactory Receptor Genes

The results of our qPCR-experiments regarding olfactory receptor genes revealed that the expression level of genes that are organized in a typical zonal pattern in the OE (*olfr15, 78* and *1507*) was unaltered in *NPC1^-/-^* mice compared to wildtype individuals. In contrast, a significant reduction of mRNA in *NPC1^-/-^* mice was found for genes that belong to the OR37 subfamily. Interestingly, neither the treatment with HPßCD nor the combination treatment could rescue this problem completely; thus, regarding these features, the OR37 gene group turned out to be different from classical ORs. The result is consistent with our previous observations that the OR37 genes display unique features, setting them apart from other ORs. They are, for example, expressed by sensory neurons that are clustered in the center of the OE and send their axons to only a single glomerulus in the OB (Strotmann et al., 2000). This is in contrast to the canonical ORs that are expressed by ORNs widely distributed throughout the epithelium and project to two or even more glomeruli. On the next level of organization - the transfer of information to higher brain centers - OR37 projection neurons have been shown not to connect to the typical olfactory cortex, but to nuclei in the amygdala and hypothalamus (Bader et al., 2012a,b), brain regions related to social phenomena or stress. Thus, the OR37 subsystem is supposed to be involved in social communication and may elicit innate reactions in mice (Klein et al., 2015). It is currently not known whether chemosensors in the nose that are involved in social communication are particularly affected in NPC1; the results of our present study indicate, however, that it may be worth addressing this question in future studies.

### Massive Glia Activation in the Olfactory Bulb of *NPC1*^-/-^

Interestingly, we found an increase in the density of BrdU(+) proliferating cells as well as a marked enhancement of apoptosis in the OB of sham-treated *NPC1*^-/-^ mice. The increased proliferation activity may be due to the increased number of microglia cells and astrocytes. This is consistent with the observation made by Seo et al. (2014) who demonstrated an increase of the neurogenic activity in 8 weeks old *NPC1*^-/-^ mice. They found a co-localization of BrdU and Iba1 in 36% of the newly formed cells, implying a 3-fold enhancement of rapidly proliferating microglia in *NPC1*^-/-^ when compared to healthy controls. Further on, they demonstrated that the excessive microgliosis contributed to a progression of olfactory impairment due to a markedly increased apoptosis and inhibited neuronal maturation (Seo et al., 2014). The role of glia in neurodegenerative processes has been controversially discussed. Although there is increasing evidence that glia activation is a result of neuronal death (Suzuki et al., 2003; Chen et al., 2007), it might also be the reason for neurodegeneration (German et al., 2002; Seo et al., 2014, 2016). The elevated numbers of Iba1(+) cells obtained in HPßCD- treated *NPC1*^-/-^ mice seem somewhat inconclusive since the western blot analysis did not reveal an upregulation.

### No Loss of Dopaminergic Neurons in *NPC1*^-/-^ Mice

Our previous studies have shown a dramatic loss of ORNs in the OE of *NPC1*^-/-^ mice (Meyer et al., 2017). Several studies described a decline of TH(+) PGs after sensory deprivation or lesion of the OE (Nadi et al., 1981; Stone et al., 1990; Baker et al., 1993; Cho et al., 1996) and thus it seemed reasonable to hypothesize that the number of TH(+) dopaminergic PG is reduced also in *NPC1*^-/-^ mice. Seo et al. (2014) indeed demonstrated a reduction of the TH(+) immunoreactivity by half in the OB of *NPC1*^-/-^ mice. Surprisingly, our quantification revealed reduced numbers of TH(+) neurons compared with *NPC1*^+/+^. However, the TH(+) immunoreactivity exhibited alterations in the TH(+) signal within the glomeruli suggesting a reduction of TH(+) nerve fibers in *NPC1*^-/-^. Western blot results, however, support the findings of Seo et al. (2014) who determined the whole signal intensity rather than the number of TH(+) cells, indicating that the reduction of the TH(+) signal in *NPC1*^-/-^ is caused by a diminution of TH protein in dopaminergic axons and dendrites rather than a destruction of cells.

### Reconstitution of Olfactory Function After Combination and HPßCD Treatment

A central finding of the present study is that the treatment of a Niemann–Pick disease mouse model with combination or HPßCD led to a significant improvement of olfactory function. While combination-treated *NPC1*^-/-^ mice needed on average about 20% longer latency time than *NPC1*^+/+^ control mice, HPßCD- treated *NPC1*^-/-^ mice were even minimally faster indicating that both therapies could normalize olfactory function in 8 weeks old *NPC1*^-/-^ mice.

Our mRNA data for *Omp* and *Adcy3* indicated no regulation of surviving ORN in *NPC1*^-/-^ mice. Also, previous electrophysiological recordings of the OE revealed no significant latencies (Hovakimyan et al., 2013b) supporting the notion that OE dynamics such as extremely increased proliferation and generation of new progenitors might compensate for the loss of ORN in sham-treated *NPC1*^-/-^ mice. Therefore, olfactory deficits are likely to be due to central deficits at the level of the OB. After treatment, OB morphology showed less micro- and astrogliosis as well as a decrease of apoptosis even though a complete normalization to the level of *NPC1*^+/+^ mice could not be realized. Former investigations of the cell and tissue dynamics of the OE of *NPC1*^-/-^ revealed a markedly reduced apoptosis and macrophage activity in combination-treated *NPC1*^-/-^ (Meyer et al., 2017).

The monotherapy with HPßCD also revealed a normalization of olfactory function and a visible reduction of apoptosis and astrogliosis in *NPC1*^-/-^mice. Several studies proved the benefit of HPßCD in mice (Liu et al., 2010; Maass et al., 2015; Tanaka et al., 2015) and humans (Ramirez et al., 2010; Maarup et al., 2015). In the *NPC1*^-/-^ OB, proliferation as well as Iba1(+) microglial activity remained unchanged after HPßCD therapy and complied with the profile of sham-treated *NPC1*^-/-^ mice. Surprisingly, both combination and HPßCD treatment did not lead to increased numbers of TH(+) dopaminergic neurons and increased protein levels in the OB, which could otherwise be linked with impaired olfactory acuity (Huisman et al., 2004; Mundinano et al., 2011).

The slight, but not significant increase of TH(+) neurons and simultaneously constant protein content after HPßCD treatment may indicate a reduction of TH protein per cell rather than a substantial change of cell numbers, as reported by Seo et al. (2014, 2016). Also, microglia activity is able to positively influence the survival of TH(+) mesencephalic neurons (Nagata et al., 1993).

## Conclusion

The present study sheds light on the issue, if easy- to- perform olfactory tests in patients with neurodegenerative diseases may be used as predictive or control tests for the course of a disease, e.g., in dependence of a treatment strategy. Our data in NPC1 show that both treatment approaches prevent neurodegeneration and simultaneously ameliorate olfactory dysfunction.

What is more, these investigations should be expanded to study not only prevention of neurodegenerative symptoms, but also their reversal after a later onset of treatment efforts, which seems more realistic in practice.

## Author Contributions

AM and MW conceived and designed the experiments. AM and AG performed the experiments. AM, AG, AB, JS, and MW analyzed the data. AM, AG, AB, MW, AR, and AW wrote the paper. All the authors read and approved the last version of the manuscript.

## Conflict of Interest Statement

The authors declare that the research was conducted in the absence of any commercial or financial relationships that could be construed as a potential conflict of interest.

## Supplementary Material

FIGURE S1 Scheme of the drug application for the combination treatment. Only mice used for immunohistochemical experiments received BrdU.

FIGURE S2 Performance of *NPC1*^+/+^ and different treated *NPC1*^-/-^ mice on surface pellet test. Mean values of the latencies vary from minimum 5.16 s (sham-treated *NPC1*^+/+^) to a maximum of 11.07 s (HPßCD-treated *NPC1*^-/-^), indicating that all tested mice most likely have no impairments of motor skills or an altered motivation for foraging. Box plot graphs represent the mean ± SEM and depict the median, the upper and lower quartiles, and outliers (pentagon and circle). ^∗^*p* ≤ 0.05, ^∗∗^*p* ≤ 0.01.

FIGURE S3 Housekeeping genes *Ppia* and *ß-Actin* were not regulated in the olfactory bulb (OB) and the olfactory epithelium (OE) of *NPC1^-/-^* mice. Determination of relative expression of certain markers and receptors require normalization to reference genes. The analyses of Cq values (cycle of quantification) resulted in *Ppia* and *ß-Actin* as appropriate housekeeping genes in the OB and OE **(A–D)**. No regulation was present between *NPC1^+/+^* and *NPC1^-/-^* mice. Data are represented as mean ± SEM, *n* = 10–15.

TABLE S1 Results of the buried and the surface pellet test. Latencies are expressed as the mean values ± SEM in s.

TABLE S2 FAM-MGB coupled Taqman probes and housekeeping genes used for quantitative RT-PCR.

TABLE S3 Results of the BrdU(+) and TH(+) quantification of the unilateral OB. Cell densities are expressed as the mean values ± SEM in cells/mm^3^.

TABLE S4 Quantitative RT-PCR displays the relative expression of *Bax* and *Bcl2* mRNA including the *Bax/Bcl2 ratio* in the olfactory bulb (OB) of differently treated *NPC1^-/-^* mice (*n* = 3) compared to *NPC1^+/+^* mice (*n* = 3). Data are normalized to *Ppia* and represent as mean ± SEM.

## Abbreviations

Adcy3: Adenylate cyclase 3
Bax: Bcl2-associated X protein
BC: Basal cells
Bcl2: B cell leukemia/lymphoma 2
DK: Cilia/dendritic knobs
EPL: external plexiform layer
GCL: granule cell layer
GL: glomerular layer
HPßCD: 2-hydroxypropyl-ß-cyclodextrin
IPL: internal plexiform layer
LP: Lamina propria
MCL: mitral cell layer
NPC1: Niemann–Pick disease type C1
OB: olfactory bulb
OE: olfactory epithelium
Olfr: olfactory receptor gene
OMP: olfactory marker protein
ONL: olfactory nerve layer
OR: olfactory receptor
ORN: olfactory receptor neuron
PG: Periglomerular
PPIA: Cyclophilin A
