# Supplementary figures and images for: Olfactory Performance as an Indicator for Protective Treatment Effects in an Animal Model of Neurodegeneration
Source: Front Integr Neurosci. 2018 Aug 14;12:35. doi: 10.3389/fnint.2018.00035 (PMC6102364; doi:10.3389/fnint.2018.00035)

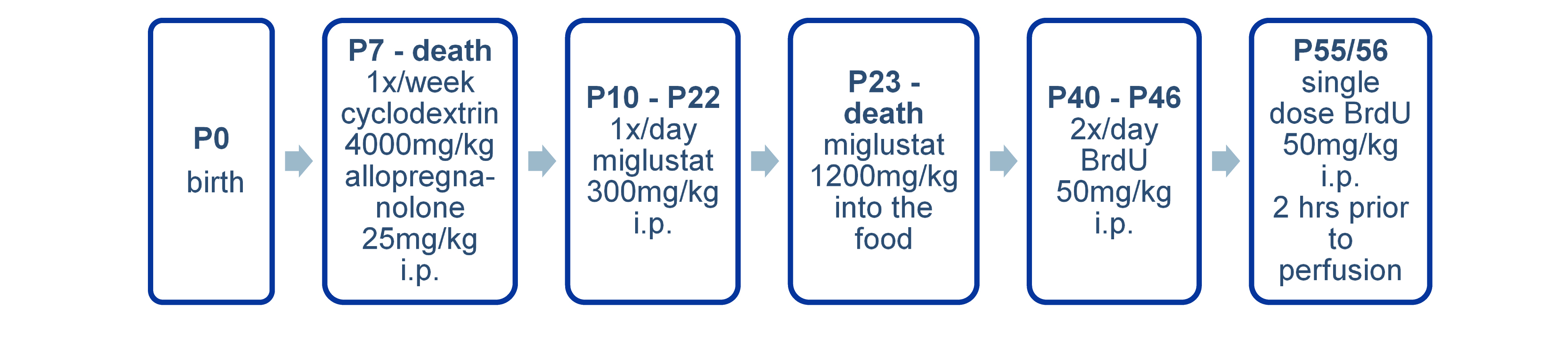

Supplement: FIGURE S1 — Scheme of the drug application for the combination treatment. Only mice used for immunohistochemical experiments received BrdU. [file Image_1.JPEG]

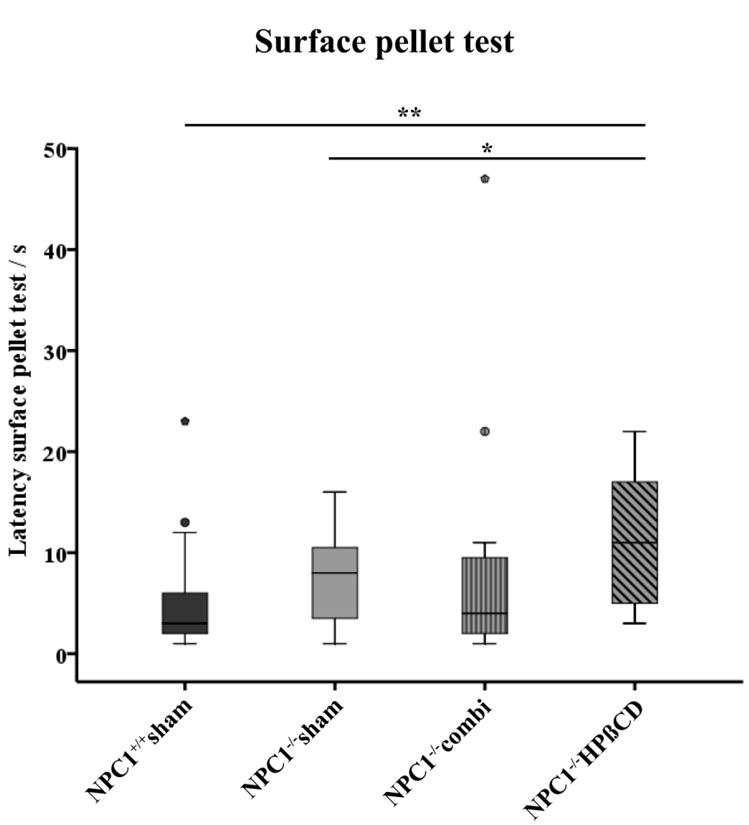

Supplement: FIGURE S2 — Performance of NPC1+/+ and different treated NPC1-/- mice on surface pellet test. Mean values of the latencies vary from minimum 5.16 s (sham-treated NPC1+/+) to a maximum of 11.07 s (HPßCD-treated NPC1-/-), indicating that all tested mice most likely have no impairments of motor skills or an altered motivation for foraging. Box plot graphs represent the mean ± SEM and depict the median, the upper and lower quartiles, and outliers (pentagon and circle). ∗p ≤ 0.05, ∗∗p ≤ 0.01. [file Image_2.JPEG]

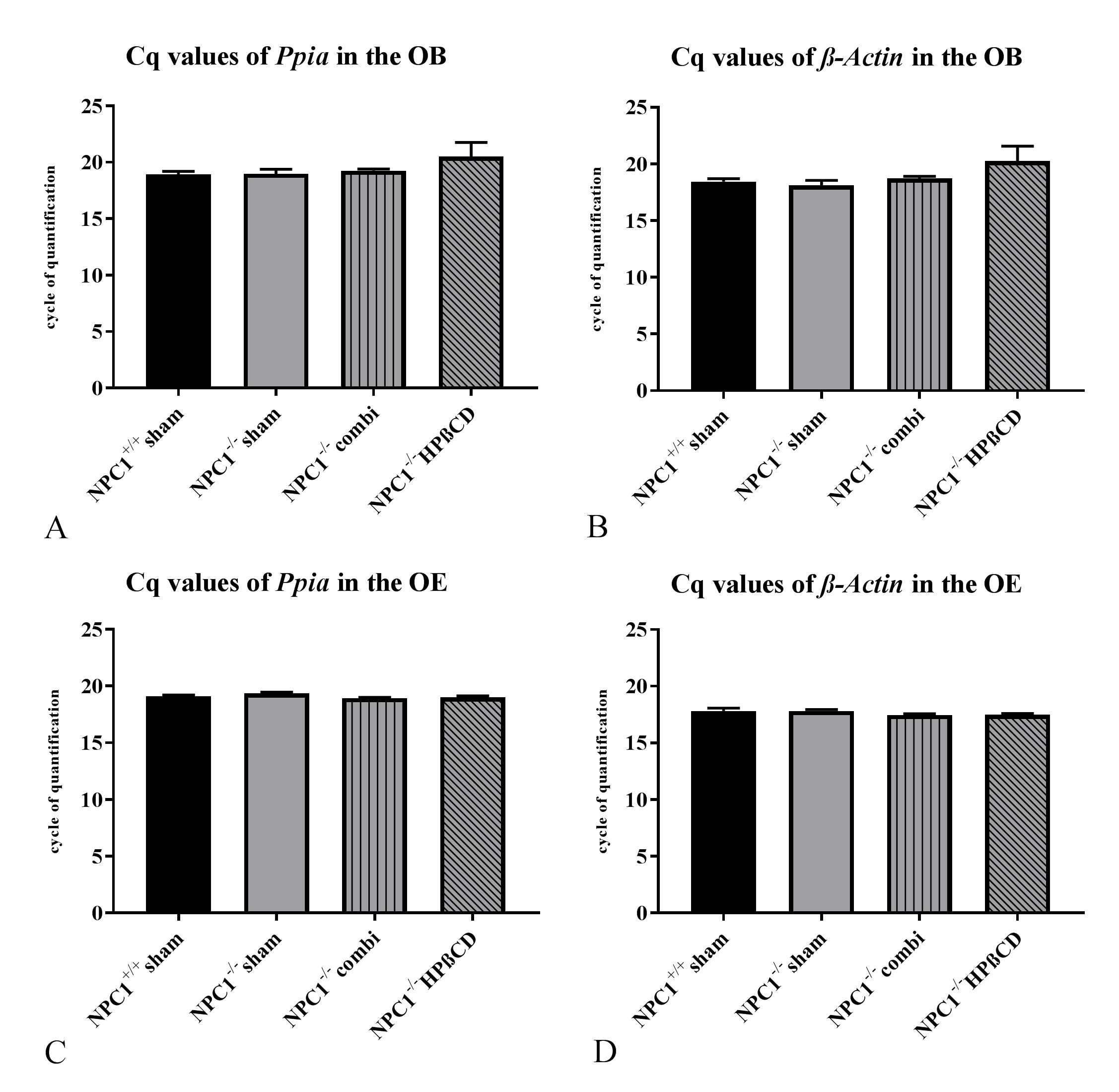

Supplement: FIGURE S3 — Housekeeping genes Ppia and ß-Actin were not regulated in the olfactory bulb (OB) and the olfactory epithelium (OE) of NPC1-/- mice. Determination of relative expression of certain markers and receptors require normalization to reference genes. The analyses of Cq values (cycle of quantification) resulted in Ppia and ß-Actin as appropriate housekeeping genes in the OB and OE (A–D). No regulation was present between NPC1+/+ and NPC1-/- mice. Data are represented as mean ± SEM, n = 10–15. [file Image_3.TIF]
