# Supplementary material for: Olfactory Performance as an Indicator for Protective Treatment Effects in an Animal Model of Neurodegeneration
Source: Front Integr Neurosci. 2018 Aug 14;12:35. doi: 10.3389/fnint.2018.00035 (PMC6102364; doi:10.3389/fnint.2018.00035)
Supplement: TABLE S1 — Results of the buried and the surface pellet test. Latencies are expressed as the mean values ± SEM in s. [file Table_1.DOCX]

Supplementary Table 1: Results of the buried and the surface pellet test. Latencies are expressed as the mean values ± SEM in s.

| **Treatment group** | **Latency buried pellet test** | | |  | **Latency surface pellet test** | | |
| --- | --- | --- | --- | --- | --- | --- | --- |
|  | **(s ± SEM)** | | |  | **(s ± SEM)** | | |
| **NPC1^+/+^ sham** | 53.32 | ± | 11.66 |  | 5.16 | ± | 1.29 |
| **NPC1^-/-^ sham** | 145.25 | ± | 27.11 |  | 7.50 | ± | 1.13 |
| **NPC1^-/-^ combi** | 64.27 | ± | 12.42 |  | 8.53 | ± | 3.11 |
| **NPC1^-/-^ HPßCD** | 47.57 | ± | 7.88 |  | 11.07 | ± | 1.80 |
