# Supplementary material for: Olfactory Performance as an Indicator for Protective Treatment Effects in an Animal Model of Neurodegeneration
Source: Front Integr Neurosci. 2018 Aug 14;12:35. doi: 10.3389/fnint.2018.00035 (PMC6102364; doi:10.3389/fnint.2018.00035)
Supplement: TABLE S2 — FAM-MGB coupled Taqman probes and housekeeping genes used for quantitative RT-PCR. [file Table_2.DOCX]

**Supplementary Table 2:** FAM-MGB coupled Taqman probes and housekeeping genes used for quantitative RT-PCR.

| **Gene** | **Assay ID** |
| --- | --- |
| *Ki67*, antigen identified by monoclonal antibody Ki 67 | Mm01278617_m1 |
| *Bax*, Bcl2-associated X protein, apoptosis regulator | Mm01205549_m1 |
| *Bcl2*, B cell leukemia/lymphoma 2, apoptosis regulator | Mm00477631_m1 |
| *Gap43*, growth associated protein 43 | Mm00500404_m1 |
| *Omp*, olfactory marker protein | Mm00448081_s1 |
| *Adcy3*, adenylate cyclase 3 | Mm00460371_m1 |
| *Olfr15*, olfactory receptor 15 | Mm00435446_s1 |
| *Olfr78*, olfactory receptor 78 | Mm00628116_m1 |
| *Olfr155*, olfactory receptor 155 | Mm00497836_s1 |
| *Olfr156*, olfactory receptor 156 | Mm03808833_sH |
| *Olfr157*, olfactory receptor 157 | Mm00497861_s1 |
| *Olfr1507*, olfactory receptor 1507 | Mm00451539_s1 |
| *Ppia*, cyclophilin A | Mm02342430_g1 |
| *Actb*, ß-actin | Mm00607939_s1 |
