# Supplementary material for: Olfactory Performance as an Indicator for Protective Treatment Effects in an Animal Model of Neurodegeneration
Source: Front Integr Neurosci. 2018 Aug 14;12:35. doi: 10.3389/fnint.2018.00035 (PMC6102364; doi:10.3389/fnint.2018.00035)
Supplement: TABLE S3 — Results of the BrdU(+) and TH(+) quantification of the unilateral OB. Cell densities are expressed as the mean values ± SEM in cells/mm3. [file Table_3.DOCX]

Supplementary Table 3: Results of the BrdU(+) and TH(+) quantification of the unilateral OB. Cell densities are expressed as the mean values ± SEM in cells/mm³.

| **Treatment group** | **BrdU**  **(cells/mm³ ± SEM)** | | |  | **TH**  **(cells/mm³ ± SEM)** | | |
| --- | --- | --- | --- | --- | --- | --- | --- |
| ***NPC1^+/+^* sham** | 77,326 | ± | 9,109 |  | 88,419 | ± | 17,605 |
| ***NPC1^-/-^* sham** | 109,557 | ± | 20,446 |  | 87,093 | ± | 5,202 |
| ***NPC1^-/-^* combi** | 71,779 | ± | 4,405 |  | 99,714 | ± | 13,380 |
| ***NPC1^-/-^* HPßCD** | 118,954 | ± | 9,298 |  | 123,252 | ± | 15,234 |
