# Supplementary material for: Olfactory Performance as an Indicator for Protective Treatment Effects in an Animal Model of Neurodegeneration
Source: Front Integr Neurosci. 2018 Aug 14;12:35. doi: 10.3389/fnint.2018.00035 (PMC6102364; doi:10.3389/fnint.2018.00035)
Supplement: TABLE S4 — Quantitative RT-PCR displays the relative expression of Bax and Bcl2 mRNA including the Bax/Bcl2 ratio in the olfactory bulb (OB) of differently treated NPC1-/- mice (n = 3) compared to NPC1+/+ mice (n = 3). Data are normalized to Ppia and represent as mean ± SEM. [file Table_4.DOCX]

**Supplementary Table 4: Quantitative RT-PCR displays the relative expression of *Bax* and *Bcl2* mRNA including the *Bax/Bcl2 ratio* in the olfactory bulb (OB) of differently treated *NPC1^-/-^* mice (n=3) compared to *NPC1^+/+^* mice (n=3).** Data are normalized to *Ppia* and represent as mean ± SEM.

|  |  | ***Bax* mRNA** | | |  | ***Bcl2* mRNA** | | |  | ***Bax/Bcl2* mRNA** | | |
| --- | --- | --- | --- | --- | --- | --- | --- | --- | --- | --- | --- | --- |
|  |  | **(Mean ± SEM)** | | |  | **(Mean ± SEM)** | | |  | **(Mean ± SEM)** | | |
| ***NPC1^+/+^* sham** |  | 0.0344 | ± | 0.0015 |  | 0.0271 | ± | 0.0023 |  | 1.2757 | ± | 0.0548 |
| ***NPC1^-/-^* sham** |  | 0.0470 | ± | 0.0030 |  | 0.0278 | ± | 0.0021 |  | 1.7154 | ± | 0.1958 |
| ***NPC1^-/-^* combi** |  | 0.0374 | ± | 0.0023 |  | 0.0309 | ± | 0.0024 |  | 1.2195 | ± | 0.0860 |
| ***NPC1^-/-^* HPßCD** | | 0.0256 | ± | 0.0043 |  | 0.0251 | ± | 0.0020 |  | 1.0085 | ± | 0.1318 |
